# Changes in STI and HIV testing and testing need among men who have sex with men during the UK’s COVID-19 pandemic response

**DOI:** 10.1136/sextrans-2022-055429

**Published:** 2022-07-21

**Authors:** Jack RG Brown, David Reid, Alison R Howarth, Hamish Mohammed, John Saunders, Caisey V Pulford, Gwenda Hughes, Catherine H Mercer

**Affiliations:** 1 UCL Institute for Global Health, University College London, London, UK; 2 The National Institute for Health Research Health Protection Research Unit in Blood Borne and Sexually Transmitted Infections at University College London in partnership with the UK Health Security Agency, London, UK; 3 Blood Safety, Hepatitis, STIs and HIV Division, UK Health Security Agency, London, UK; 4 UK Public Health Rapid Support Team, London School of Hygiene and Tropical Medicine Department of Infectious Disease Epidemiology, London, UK

**Keywords:** sexual behaviour, sexual health, HIV, COVID-19

## Abstract

**Objectives:**

We examined the impact of COVID-19-related restrictions on sexual behaviours, STI and HIV testing and testing need among men who have sex with men (MSM) in the UK.

**Methods:**

We used social media and dating applications to recruit to three cross-sectional surveys (S1–S3) during the UK’s pandemic response (S1: 23 June–14 July 2020; S2: 23 November–12 December 2020; S3: 23 March–14 April 2021). Surveys included lookback periods of around 3–4 months (P1–P3, respectively). Eligible participants were UK resident men (cisgender/transgender) and gender-diverse people assigned male at birth (low numbers of trans and gender-diverse participants meant restricting these analyses to cisgender men), aged ≥16 years who reported sex with men (cisgender/transgender) in the last year (S1: N=1950; S2: N=1463; S3: N=1487). Outcomes were: recent STI/HIV testing and unmet testing need (new male and/or multiple condomless anal sex partners without a recent STI/HIV test). Crude and adjusted associations with each outcome were assessed using logistic regression.

**Results:**

Participants’ sociodemographic characteristics were similar across surveys. The proportion reporting a recent STI and/or HIV test increased between P1 and P2 (25.0% to 37.2% (p<0.001) and 29.7% to 39.4% (p<0.001), respectively), then stabilised in P3 (40.5% reporting HIV testing). Unmet STI testing need increased across P1 and P2 (26.0% to 32.4%; p<0.001), but trends differed between groups, for example, unmet STI testing need was higher in bisexually-identifying (vs gay-identifying) MSM across periods (adjusted OR (aOR): P1=1.64; P2=1.42), but declined in HIV-positive (vs HIV-negative/unknown) MSM (aOR: P1=2.06; P2=0.68). Unmet HIV testing need increased across P1 and P2 (22.9% to 31.0%; p<0.001) and declined in P3 (25.1%; p=0.001). During P3, MSM reporting a low life-satisfaction level (vs medium–very high) had greater unmet need (aOR: 1.44), while from P2 onwards HIV pre-exposure prophylaxis users (vs non-users) had lower unmet need (aOR: P2=0.32; P3=0.50).

**Conclusion:**

Considerable unmet STI/HIV testing need occurred among MSM during COVID-19-related restrictions, especially in bisexually-identifying men and those reporting low life satisfaction. Improving access to STI/HIV testing in MSM is essential to prevent inequalities being exacerbated.

**Key messages**

- There is currently little evidence on how the UK’s fluctuating social restrictions in response to COVID-19 have impacted men who have sex with men (MSM)’s sexual behaviour and health.
- From three large, community-based surveys of MSM, we identified factors associated with STI/HIV testing and testing need over the first year of the UK’s COVID-19 response.
- A sizeable portion of MSM reported STI/HIV risk during restrictions and unmet testing need was disproportionately found among groups who already experience poor sexual health.
- These data complement national clinical data and help inform future sexual health policy and service delivery to address inequalities as COVID-19 restrictions have been eased.

## Introduction

In the UK, gay, bisexual and other men who have sex with men (MSM) bear a disproportionate burden of STIs including HIV. All sexually active MSM are advised to test for STIs and HIV annually.^1^ Those practising behaviours with increased STI/HIV risk (referred hereafter for brevity as ‘sexual risk behaviour’), for example, HIV pre-exposure prophylaxis (PrEP) users and those reporting multiple recent condomless anal sex (CAS) partners, are recommended to test quarterly.^1^

On 23 March 2020, the UK announced its first national lockdown in response to rising SARS-CoV-2 (COVID-19) diagnoses (online supplemental appendix 1).^2^ Consequently, sexual health services (SHS) rapidly reconfigured: in-person asymptomatic screening and walk-in appointments were suspended and patients directed online.^3^ While the introduction of social restrictions during the first national lockdown^4 5^ led to reductions in sexual risk behaviours for MSM (eg, one UK study of MSM found 47% reported new recent sex partners March–June/July 2020, a substantial decrease from 71% in a comparative 2017 sample),^6^ they continued to be reported by a high proportion.^6–8^

10.1136/sextrans-2022-055429.supp1 Supplementary data

Social restrictions eased July–September 2020,^9^ and sexual risk behaviour among MSM seemed to increase compared with the first national lockdown. A London-based study including MSM found 34% reported physical sexual contact March–April/May 2020,^10^ increasing to 68% by August–November 2020, among whom 71% reported sex with casual partners outside their household.^11^ From September 2020 onwards, restrictions were gradually re-introduced until a second national lockdown during November 2020. A further brief easing of national restrictions during December was followed by a third national lockdown January–March 2021, whereafter restrictions were gradually eased leading to full removal from late July.

It is important to understand how these rapid changes in social restrictions influenced sexual behaviours as well as STI/HIV testing and need, especially in the context of the reconfigured SHS. We used data from large, community-based cross-sectional surveys conducted across three periods between March 2020–April 2021 to identify factors associated with STI and HIV testing and testing need among MSM over the first year of the UK’s COVID-19 pandemic response.

## Methods

### Study design

The ‘Reducing inequalities in Sexual Health’ (RiiSH)-COVID surveys are repeat, cross-sectional online community surveys, each fielded for 2–3 weeks during different stages of the pandemic (online supplemental appendix 1): 23 June–14 July 2020 (survey (S)1); then 23 November–12 December 2020 (S2); and 23 March–14 April 2021 (S3).

### Setting and sampling

Participants were recruited from social networking sites (Facebook, Twitter, Instagram) and geospatial dating applications (Grindr: S1–S3; Hornet: S1–S2). Adverts on these sites and applications directed individuals to the anonymous online survey. The first questions assessed eligibility, defined as: UK resident; aged ≥16 years; men (cisgender/transgender), transwomen or gender-diverse people assigned male at birth (AMAB); reporting sex in the past year with a man (cisgender/transgender) or gender-diverse person AMAB. The survey took on average 10 minutes to complete. Online consent was obtained from all participants. No financial incentive was offered.

### Data collection

The RiiSH-COVID survey was adapted from a survey conducted in 2017^12 13^ and was administered using SNAPSurvey software. The survey included questions on STI/HIV testing, PrEP use, SHS use, sexual relationships and behaviour, use of chemsex drugs (crystal methamphetamine, mephedrone, gamma-hydroxybutyrate/gamma-butyrolactone), personal well-being (using the Office for National Statistics’ well-being measures^14^) and COVID-19 experience (eg, infection, testing and self-reported symptoms). Questions about the last occurrence of behaviours referred to lookback periods which related to around 3–4 months prior to the survey. These lookback periods (P1–P3, for S1–S3, respectively) roughly correspond to:

- P1: from the beginning of the first national lockdown (23 March 2020) until June/July 2020.
- P2: from when the first lockdown restrictions were eased to minimal restrictions (July 2020) until November/December 2020.
- P3: from the beginning of the third national lockdown (from late December 2020) until March/April 2021.

The questionnaires are given in online supplemental appendices 2a, 2b and 2c.

10.1136/sextrans-2022-055429.supp2 Supplementary data

10.1136/sextrans-2022-055429.supp3 Supplementary data

10.1136/sextrans-2022-055429.supp4 Supplementary data

### Data analysis

The data were checked and 14 duplicate entries removed, leaving a total of 5066 participants (S1: N=2018; S2: N=1522; S3: N=1526). The analyses were restricted to data from 4900 cisgender MSM participants (S1: N=1950; S2: N=1463; S3: N=1487), as few transgender and gender-diverse people AMAB participated. The denominator for the HIV testing analyses was further restricted to cisgender MSM reporting a HIV-negative/unknown status (S1: N=1753; S2: N=1308; S3: N=1330). Due to relatively small numbers of participants from ethnic minority groups, we grouped participants by whether they identified as white or not (hereafter: ‘all other ethnic groups’). We did not collect data on STI testing in S3, therefore, analyses of this outcome are limited to S1 and S2. STATA V.16.1 was used for analyses.

Pearson’s χ^2^ test was used to examine differences in proportions between surveys in sociodemographic, health, sexual behavioural factors and the two primary outcomes, each considered separately for STIs and HIV:

- Accessing testing, defined as reporting a test in the lookback period either in-person or through an online self-sample testing service.
- Unmet testing need, defined as reporting one or more new sex partners and/or multiple CAS partners in the lookback period without testing during that period.^1^

Binary logistic regression was used to examine associations between sociodemographic, health and sexual behavioural variables and these outcomes. Explanatory variables that were statistically significant (p<0.05) in binary regression were included in multivariable logistic regression models to identify independent associations. We re-ran the multivariable regression models including interaction terms to assess whether the magnitude of the effect of an explanatory variable on the outcome changed significantly across surveys.

## Results

### Participants’ characteristics

In each survey, over half of participants were recruited via a dating application (S1=53.0%; S2=62.3%; S3=58.5%; p<0.001), with the remainder recruited through social media. There was little difference across surveys in the profile of participants (table 1; online supplemental appendix 3). Participants had a median age of 40 years (IQR: 30–51; range: 16–81) across surveys. The majority identified as white (88.8%), gay (84.7%), resident in England (84.9%), with around three-quarters (77.9%) born in the UK. More than half (57.5%) reported having a degree, and a majority (76.6%) reported having some form of employment, including those on ‘furlough’ (where the UK government paid 80% of the salary of those unable to work due to COVID-19 restrictions).^15^ Around one-third (35.2%) of participants lived alone and another third (31.1%) lived with their partner(s). One in 10 (10.4%) participants reported living with HIV.

**Table 1:** Sociodemographic characteristics, health-related factors and sexual behaviours associated with reporting testing for STIs during two periods of the first year of the UK’s pandemic response

Lookback period (period number)	March–June/July 2020 (P1)						July–November/December 2020 (P2)					
	Sample composition, col % (n)	(Row) % reporting a recent STI test (n)	uOR (95% CI) for reporting a recent STI test	P value	aOR (95% CI)* for reporting a recent STI test	P value	Sample composition, col % (n)	(Row) % reporting a recent STI test (n)	uOR (95% CI) for reporting a recent STI test	P value	aOR (95% CI)* for reporting a recent STI test	P value
All†	100.0 (1950)	25.0 (487)	–	–	–	–	100.0 (1463)	37.2 (544)	–	–	–	–
**Sociodemographic characteristics**												
Age (years)												
Under 30	24.6 (479)	28.8 (138)	1	<0.001	1	0.001	26.5 (388)	39.2 (152)	1	0.005	1	0.136
30–44	35.3 (688)	28.3 (195)	0.98 (0.76 to 1.27)		0.82 (0.61 to 1.10)		37.8 (553)	40.9 (226)	1.07 (0.82 to 1.40)		0.79 (0.57 to 1.09)	
45 and over	40.2 (783)	19.7 (154)	0.60 (0.46 to 0.79)		0.58 (0.43 to 0.79)		35.7 (522)	31.8 (166)	0.72 (0.55 to 0.95)		0.71 (0.50 to 1.00)	
Sexual identity												
Gay	86.1 (1678)	26.5 (444)	1	<0.001	1	0.003	83.4 (1220)	39.3 (479)	1	<0.001	1	0.235
Bisexual‡	14.0 (272)	15.8 (43)	0.52 (0.37 to 0.74)		0.58 (0.40 to 0.83)		16.6 (243)	26.8 (65)	0.56 (0.42 to 0.77)		0.80 (0.56 to 1.15)	
Ethnicity												
White§	88.6 (1728)	24.5 (424)	1	0.219	–	n.a.	87.8 (1284)	36.7 (471)	1	0.290	–	n.a.
All other ethnic groups¶	11.4 (222)	28.4 (63)	1.22 (0.89 to 1.66)		–		12.2 (179)	40.8 (73)	1.19 (0.86 to 1.64)		–	
Country of residence in the UK												
England	86.1 (1679)	27.0 (454)	1	<0.001	1	<0.001	84.3 (1233)	39.9 (491)	1	<0.001	1	<0.001
Outside England	13.9 (271)	12.2 (33)	0.37 (0.26 to 0.55)		0.37 (0.25 to 0.55)		15.7 (230)	23.0 (53)	0.45 (0.33 to 0.63)		0.53 (0.36 to 0.78)	
Born in the UK												
Yes	78.1 (1523)	24.2 (368)	1	0.121	1	0.588	76.9 (1125)	33.9 (381)	1	<0.001	1	0.003
No	21.9 (427)	27.9 (119)	1.21 (0.95 to 1.54)		0.93 (0.71 to 1.22)		23.1 (338)	48.2 (163)	1.82 (1.42 to 2.33)		1.58 (1.17 to 2.14)	
Highest educational qualification												
Degree or higher	59.0 (1149)	27.1 (311)	1	0.011	1	0.065	57.4 (840)	42.0 (353)	1	<0.001	1	0.029
Below degree	41.1 (800)	22.0 (176)	0.76 (0.61 to 0.94)		0.80 (0.64 to 1.01)		42.6 (623)	30.7 (191)	0.61 (0.49 to 0.76)		0.75 (0.57 to 0.97)	
Employed (inc. furlough**)												
Yes	77.2 (1497)	25.3 (378)	1	0.656	–	n.a.	74.9 (1095)	37.9 (415)	1	0.327	–	n.a.
No	22.8 (442)	24.2 (107)	0.95 (0.74 to 1.21)		–		25.2 (368)	35.1 (129)	0.88 (0.69 to 1.13)		–	
**Health-related factors**												
HIV status												
Negative/Unknown	89.9 (1753)	25.2 (442)	1	0.462	1	0.753	89.4 (1308)	36.0 (471)	1	0.008	1	<0.001
Positive	10.1 (197)	22.8 (45)	0.88 (0.62 to 1.25)		1.06 (0.72 to 1.56)		10.6 (155)	47.1 (73)	1.58 (1.13 to 2.21)		2.88 (1.94 to 4.28)	
PrEP use (*in the lookback period*)												
No	86.4 (1677)	19.9 (333)	1	<0.001	1	<0.001	77.0 (1125)	25.2 (284)	1	<0.001	1	<0.001
Yes	13.6 (264)	57.2 (151)	5.39 (4.11 to 7.08)		4.23 (3.10 to 5.76)		23.1 (337)	77.2 (260)	10.0 (7.50 to 13.33)		7.83 (5.65 to 10.86)	
Life satisfaction level												
Medium–very high	68.8 (1340)	24.4 (327)	1	0.368	–	n.a.	75.6 (1106)	37.1 (410)	1	0.875	–	n.a.
Low	31.2 (608)	26.3 (160)	1.11 (0.89–1.38)		–		24.4 (357)	37.5 (134)	1.02 (0.80 to 1.31)		–	
**Sexual behaviour (** ***in the lookback period*** **)**												
No. of CAS partners												
None	62.1 (1211)	18.9 (229)	1	<0.001	1	<0.001	43.2 (632)	21.0 (133)	1	<0.001	1	<0.001
One	20.4 (398)	27.9 (111)	1.66 (1.28 to 2.16)		1.66 (1.24 to 2.20)		23.7 (346)	30.6 (106)	1.66 (1.23 to 2.23)		1.50 (1.07 to 2.10)	
Multiple	17.5 (341)	43.1 (147)	3.25 (2.51 to 4.21)		2.13 (1.58 to 2.87)		33.2 (485)	62.9 (305)	6.36 (4.87 to 8.29)		3.44 (2.54 to 4.65)	

*Adjusting for: age; sexual identity; country of residence; born in the UK; education; living with partner; HIV status; PrEP use; CAS.

†Cisgender MSM.

‡Including ‘Bisexual’ (S1: n=220; S2: n=179); ‘Other’ (S1: n=44; S2: n=50); ‘Straight’ (S1: n=8; S2: n=14).

§Including ‘White British’ (S1: n=1426; S2: n=1045); ‘White Irish’ (S1: n=75; S2: n=55); ‘White other’ (S1: n=227; S2: n=184).

¶Including ‘Black’ (S1: n=31; S2: n=41); ‘Asian’ (S1: n=101; S2: n=63); ‘Mixed or other’ (S1: n=90; S2: n=75).

**The UK government paid 80% of the salary of those who were unable to work due to COVID-19 restrictions.^15^

aOR, adjusted OR; CAS, condomless anal sex; CI, confidence interval; MSM, men who have sex with men; n.a., not applicable; PrEP, pre-exposure prophylaxis; uOR, unadjusted OR.

**Table 2:** Sociodemographic, health-related factors and sexual behaviours associated with reporting testing for HIV during three periods of the first year of the UK’s pandemic response

Lookback period (period number)	March–June/July 2020 (P1)						July–November/December 2020 (P2)						December 2020–March/April 2021 (P3)					
	Sample composition, col % (n)	(Row) % reporting a recent HIV test (n)	uOR (95% CI) for reporting a recent HIV test (n)	P value	aOR (95% CI)* for reporting a recent HIV test (n)	P value	Sample composition, col % (n)	(Row) % reporting a recent HIV test (n)	uOR (95% CI) for reporting a recent HIV test (n)	P value	aOR (95% CI)* for reporting a recent HIV test (n)	P value	Sample composition, col % (n)	(Row) % reporting a recent HIV test (n)	uOR (95% CI) for reporting a recent HIV test (n)	P value	aOR (95% CI)* for reporting a recent HIV test (n)	P value
All†	100.0 (1753)	29.7 (521)	–	–	–	–	100.0 (1308)	39.4 (515)	–	–	–	–	100.0 (1330)	40.5 (538)	–	–	–	–
**Sociodemographic characteristics**																		
Age (years)																		
Under 30	26.7 (468)	35.3 (165)	1	<0.001	1	<0.001	28.9 (378)	41.0 (155)	1	<0.001	1	0.311	25.8 (343)	44.6 (153)	1	<0.001	1	0.024
30–44	35.5 (623)	32.6 (203)	0.89 (0.69 to 1.14)		0.77 (0.58 to 1.01)		37.7 (493)	44.6 (220)	1.16 (0.88 to 1.52)		0.95 (0.68 to 1.32)		35.1 (466)	45.9 (214)	1.05 (0.80 to 1.40)		0.97 (0.71 to 1.33)	
45 and over	37.8 (662)	23.1 (153)	0.55 (0.42 to 0.72)		0.54 (0.40 to 0.72)		33.4 (437)	32.0 (140)	0.68 (0.51 to 0.90)		0.77 (0.54 to 1.10)		39.1 (520)	32.9 (171)	0.61 (0.46 to 0.81)		0.68 (0.49 to 0.94)	
Sexual identity																		
Gay	85.1 (1492)	31.4 (469)	1	<0.001	1	0.002	82.4 (1078)	42.0 (453)	1	<0.001	1	0.086	83.4 (1109)	42.6 (472)	1	<0.001	1	0.036
Bisexual‡	14.9 (261)	19.9 (52)	0.54 (0.39 to 0.75)		0.59 (0.42 to 0.82)		17.6 (230)	27.0 (62)	0.51 (0.37 to 0.70)		0.72 (0.50 to 1.05)		16.6 (221)	29.9 (66)	0.57 (0.42 to 0.78)		0.69 (0.49 to 0.98)	
Ethnicity																		
White§	88.5 (1552)	29.6 (460)	1	0.836	–	n.a.	87.2 (1141)	39.3 (448)	1	0.833	–	n.a.	90.0 (1197)	40.3 (482)	1	0.683	–	n.a.
All other ethnic groups¶	11.5 (201)	30.4 (61)	1.03 (0.75 to 1.42)		–		12.8 (167)	40.1 (67)	1.04 (0.74 to 1.44)		–		10.0 (133)	42.1 (56)	1.08 (0.75 to 1.55)		–	
Country of residence in the UK																		
England	85.5 (1499)	31.1 (466)	1	0.002	1	0.009	83.4 (1091)	41.9 (457)	1	<0.001	1	0.008	83.4 (1109)	41.7 (462)	1	0.043	1	0.114
Outside England	14.5 (254)	21.7 (55)	0.61 (0.45 to 0.84)		0.64 (0.46 to 0.90)		16.6 (217)	26.7 (58)	0.51 (0.37 to 0.70)		0.60 (0.41 to 0.88)		16.6 (221)	34.4 (76)	0.73 (0.54 to 0.99)		0.76 (0.54 to 1.07)	
Born in the UK																		
Yes	78.3 (1373)	29.0 (398)	1	0.205	1	0.742	76.3 (998)	34.9 (348)	1	<0.001	1	<0.001	78.5 (1044)	38.2 (399)	1	0.002	1	0.156
No	21.7 (380)	32.4 (123)	1.17 (0.92–1.50)		0.96 (0.73–1.25)		23.7 (310)	53.9 (167)	2.18 (1.68 to 2.82)		1.95 (1.42 to 2.67)		21.5 (286)	48.6 (139)	1.53 (1.17 to 1.99)		1.24 (0.92 to 1.68)	
Highest educational qualification																		
Degree or higher	58.9 (1031)	30.8 (317)	1	0.268	1	0.791	58.8 (769)	43.8 (337)	1	<0.001	1	0.199	56.2 (748)	44.1 (330)	1	0.002	1	0.223
Below degree	41.2 (721)	28.3 (204)	0.89 (0.72–1.10)		0.97 (0.77–1.22)		41.2 (539)	33.0 (178)	0.63 (0.50 to 0.79)		0.83 (0.63 to 1.10)		43.8 (582)	35.7 (208)	0.70 (0.56 to 0.88)		0.85 (0.66 to 1.10)	
Employed (inc. furlough**)																		
Yes	77.7 (1355)	30.2 (409)	1	0.549	–	n.a.	75.4 (986)	40.1 (395)	1	0.372	–	n.a.	78.0 (1037)	41.6 (431)	1	0.119	–	n.a.
No	22.3 (388)	28.6 (111)	0.93 (0.72–1.19)		–		24.6 (322)	37.3 (120)	0.89 (0.69 to 1.15)		–		22.0 (293)	36.5 (107)	0.81 (0.62 to 1.06)		–	
**Health-related factors**																		
PrEP use (*in the lookback period*)																		
No	84.9 (1481)	24.4 (362)	1	<0.001	1	<0.001	74.2 (970)	25.4 (246)	1	<0.001	1	<0.001	76.8 (1022)	29.5 (301)	1	<0.001	1	<0.001
Yes	15.1 (264)	59.5 (157)	4.54 (3.45 to 5.96)		3.56 (2.62 to 4.83)		25.8 (337)	79.8 (269)	11.64 (8.60 to 15.76)		7.70 (5.51 to 10.76)		23.2 (308)	77.0 (237)	8.00 (5.94 to 10.76)		5.71 (4.12 to 7.91)	
Life satisfaction level																		
Medium–very high	69.0 (1208)	29.1 (351)	1	0.382	–	n.a.	75.5 (988)	38.8 (383)	1	0.430	–	n.a.	75.2 (998)	41.5 (414)	1	0.223	–	n.a.
Low	31.0 (543)	31.1 (169)	1.10 (0.88 to 1.37)		–		24.5 (320)	41.3 (132)	1.12 (0.86 to 1.43)		–		24.8 (329)	37.7 (124)	0.85 (0.66–1.10)		–	
**Sexual behaviour (** ***in the lookback period*** **)**																		
No. of CAS partners																		
None	63.0 (1105)	23.3 (257)	1	<0.001	1	<0.001	43.8 (573)	23.2 (133)	1	<0.001	1	<0.001	50.0 (665)	30.2 (201)	1	<0.001	1	<0.001
One	20.8 (364)	33.8 (123)	1.68 (1.30 to 2.18)		1.64 (1.24 to 2.17)		24.9 (325)	36.6 (119)	1.91 (1.42 to 2.57)		1.77 (1.26 to 2.49)		24.7 (329)	37.4 (123)	1.38 (1.04 to 1.82)		1.21 (0.89 to 1.64)	
Multiple	16.2 (284)	49.7 (141)	3.25 (2.48 to 4.27)		2.07 (1.52 to 2.81)		31.4 (410)	64.2 (263)	5.92 (4.47 to 7.83)		3.00 (2.16 to 4.17)		25.3 (336)	63.7 (214)	4.05 (3.07 to 5.34)		2.10 (1.52 to 2.89)	

*Adjusting for: age; sexual identity; country of residence; born in the UK; education; living with partner; PrEP use; CAS.

†Cisgender MSM that report a negative or unknown HIV status.

‡Including ‘Bisexual’ (S1: n=210; S2: n=169; S3: n=171); ‘Other’ (S1: n=43; S2: n=47; S3: n=44); ‘Straight’ (S1: n=8; S2: n=14; S3: n=6).

§Including ‘White British’ (S1: n=1288; S2: n=924; S3: n=1000); ‘White Irish’ (S1: n=64; S2: n=50; S3: n=42); ‘White other’ (S1: n=200; S2: n=167; S3: n=155).

¶Including ‘Black’ (S1: n=24; S2: n=38; S3: n=22); ‘Asian’ (S1: n=98; S2: n=61; S3: n=52); ‘Mixed or other’ (S1: n=79; S2: n=68; S3: n=59).

**The UK government paid 80% of the salary of those who were unable to work due to COVID-19 restrictions.^15^

aOR, adjusted OR; CAS, condomless anal sex; CI, confidence interval; MSM, men who have sex with men; n.a., not applicable; PrEP, pre-exposure prophylaxis; uOR, unadjusted OR.

### Changes in well-being, PrEP use and sexual behaviour over time

Around one-third (31.2%) of MSM reported a low level of life satisfaction in P1. This decreased to one-quarter (24.4%) during P2 and remained at this level during P3 (24.7%, p<0.001) (tables 1 and 2; online supplemental appendix 3). Reported PrEP use in the lookback period increased from 13.6% during P1 to 23.1% and 20.7% in P2 and P3 (p<0.001), respectively.

Reporting ≥1 new physical sex partners increased between P1 and P2 (37.1% to 61.7%), with a slight drop in P3 (51.8%, p<0.001) (online supplemental appendix 4). Similar trends were seen for other risk behaviours, for example, reporting ≥5 new physical sex partners (P1: 8.1%; P2: 21.8%; P3: 14.4%, p<0.001); multiple CAS partners (P1: 17.5%; P2: 33.2%; P3: 27.6%, p<0.001); and use of chemsex drugs (P1: 3.7%; P2: 6.0%; P3: 3.8%, p=0.002).

### Changes in STI/HIV testing over time

Among all participants, 25.0% reported STI testing in P1, increasing to 37.2% by P2 (p<0.001) (table 1; online supplemental appendix 5). Among those reporting a recent STI test in P1, around one-third (34.5%) tested in-person at a healthcare facility, with this almost doubling (69.9%) in P2. Among HIV-negative/unknown participants, 29.7% reported recent HIV testing in P1, increasing to 39.4% during P2 and 40.5% during P3 (p<0.001) (table 2; online supplemental appendix 5). Among those reporting recent HIV testing in P1, only one-quarter (26.3%) tested in-person in a healthcare facility, compared with 53.8% in P2 and 44.6% in P3 (online supplemental appendix 5).

### STI testing

Adjusting for variables associated with recent STI testing, we found that in P1, bisexually-identifying (vs gay-identifying) MSM were less likely to report recent testing (aOR: 0.58 (95% CI 0.40 to 0.83), p<0.001). MSM living in UK countries outside England (vs those living in England) were less likely to test during both P1 (aOR: 0.37 (95% CI 0.25 to 0.55), p<0.001) and P2 (aOR: 0.53 (95% CI 0.36 to 0.78), p<0.001). In contrast, PrEP users (vs non-PrEP users) were more likely to test during P1 (aOR: 4.23 (95% CI 3.10 to 5.76), p<0.001) and P2 (aOR: 7.83 (95% CI 5.65 to 10.86), p<0.001). HIV-positive (vs HIV-negative/unknown) MSM were also more likely to test during P2 (aOR: 2.88 (95% CI 1.94 to 4.28), p<0.001), but not during P1.

When an interaction term for survey period was included, the likelihood of STI testing was significantly greater in P2 versus P1 for those born outside the UK (aOR: 1.58 (95% CI 1.06 to 2.34), p=0.023); living with HIV (aOR: 2.58 (95% CI 1.53 to 4.34), p<0.001); using PrEP (aOR: 1.90 (95% CI 1.25 to 2.88), p=0.003) and reporting multiple CAS partners (aOR: 2.20 (95% CI 1.47 to 3.31), p<0.001).

### HIV testing

Adjusting for variables associated with recent HIV testing, we found trends largely followed those of STI testing. Bisexually-identifying (vs gay-identifying) MSM were less likely to test for HIV during both P1 (aOR: 0.59 (95% CI 0.42 to 0.82), p=0.002) and P3 (aOR: 0.69 (95% CI 0.49 to 0.98), p=0.036). PrEP users (vs non-PrEP users) were more likely to test during P1 (aOR: 3.56 (95% CI 2.62 to 4.83), p<0.001), P2 (aOR: 7.70 (95% CI 5.51 to 10.76), p<0.001) and P3 (aOR: 5.71 (95% CI 4.12 to 7.91), p<0.001).

When an interaction term for survey period was included, the likelihood of HIV testing was significantly greater in P2 versus P1 for those born outside the UK (aOR: 1.98 (95% CI 1.32 to 2.96), p=0.001); using PrEP (aOR: 2.66 (95% CI 1.74 to 4.07), p<0.001) and reporting multiple CAS partners (aOR: 1.89 (95% CI 1.26 to 2.83), p=0.002). The likelihood of HIV testing was significantly greater in P3 versus P2 for participants reporting PrEP use (aOR: 1.91 (95% CI 1.25 to 2.91), p=0.003).

### Unmet STI testing need

Among all participants, 26.0% had unmet STI testing need during P1, increasing to 32.4% during P2 (p<0.001) (table 3).

**Table 3:** Sociodemographic characteristics and health-related factors associated with having unmet STI testing need during two periods of the first year of the UK’s pandemic response

Lookback period (period number)	March–June/July 2020 (P1)						July–November/December 2020 (P2)					
	Sample composition, col % (n)	(Row) % have unmet STI testing need (n)	uOR (95% CI) have unmet STI testing need	P value	aOR (95% CI)* have unmet STI testing need	P value	Sample composition, col % (n)	Row % have unmet STI testing need (n)	uOR (95% CI) have unmet STI testing need	P value	aOR (95% CI)* have unmet STI testing need	P value
All†	100.0 (1950)	26.0 (499)	–	–	–		100.0 (1463)	32.4 (474)	–	–	–	–
Sociodemographic characteristics												
Age (years)												
Under 30	24.6 (479)	25.3 (121)	1	0.001	1	0.534	26.5 (388)	36.9 (143)	1	0.094	1	0.378
30–44	35.3 (688)	27.5 (189)	1.12 (0.86 to 1.46)		1.02 (0.78 to 1.35)		37.8 (553)	30.7 (170)	0.76 (0.58 to 1.00)		0.86 (0.64 to 1.15)	
45 and over	40.2 (783)	24.1 (189)	0.94 (0.72 to 1.22)		0.90 (0.68 to 1.18)		35.7 (522)	30.8 (161)	0.76 (0.58 to 1.01)		0.82 (0.61 to 1.09)	
Sexual identity												
Gay	86.1 (1678)	24.3 (408)	1	0.002	1	0.001	83.4 (1220)	30.5 (372)	1	0.001	1	0.019
Bisexual‡	14.0 (272)	33.5 (91)	1.56 (1.19 to 2.06)		1.64 (1.23 to 2.18)		16.6 (243)	42.0 (102)	1.65 (1.24 to 2.19)		1.42 (1.06 to 1.90)	
Ethnicity												
White§	88.6 (1728)	24.8 (428)	1	0.023	1	0.023	87.8 (1284)	32.2 (414)	1	0.733	1	0.734
All other ethnic groups¶	11.4 (222)	32.0 (71)	1.43 (1.06 to 1.93)		1.44 (1.05 to 1.98)		12.2 (179)	33.5 (60)	1.06 (0.76 to 1.48)		1.06 (0.75 to 1.51)	
Country of residence in the UK												
England	86.1 (1679)	24.3 (408)	1	0.002	1	<0.001	84.3 (1233)	31.0 (382)	1	0.008	1	0.056
Outside England	13.9 (271)	33.6 (91)	1.57 (1.20 to 2.07)		1.71 (1.29 to 2.27)		15.7 (230)	40.0 (92)	1.49 (1.11 to 1.99)		1.34 (0.99 to 1.81)	
Born in the UK												
Yes	78.1 (1523)	25.1 (382)	1	0.335	–	n.a.	76.9 (1125)	32.8 (369)	1	0.549	–	n.a.
No	21.9 (427)	27.4 (117)	1.13 (0.88–1.44)		–		23.1 (338)	31.1 (105)	0.92 (0.71 to 1.20)		–	
Highest educational qualification												
Degree or higher	59.0 (1149)	25.2 (290)	1	0.660	–	n.a.	57.4 (840)	30.5 (256)	1	0.068	–	n.a.
Below degree	41.1 (800)	26.1 (209)	1.05 (0.85 to 1.29)		–		42.6 (623)	35.0 (218)	1.23 (0.98 to 1.53)		–	
Employed (inc. furlough**)												
Yes	77.2 (1497)	26.9 (403)	1	0.012	1	0.012	74.9 (1095)	31.8 (348)	1	0.385	1	0.884
No	22.8 (442)	21.0 (93)	0.72 (0.56 to 0.93)		0.71 (0.55 to 0.93)		25.2 (368)	34.2 (126)	1.12 (0.87 to 1.44)		1.02 (0.78 to 1.33)	
Health-related factors												
HIV status												
Negative/Unknown	89.9 (1753)	24.5 (429)	1	0.001	1	<0.001	89.4 (1308)	33.0 (432)	1	0.130	1	0.048
Positive	10.1 (197)	35.5 (70)	1.70 (1.25 to 2.32)		2.06 (1.48 to 2.86)		10.6 (155)	27.1 (42)	0.75 (0.52 to 1.09)		0.68 (0.46 to 1.00)	
PrEP use (*in the lookback period*)												
No	86.4 (1677)	25.4 (426)	1	0.607	1	0.275	77.0 (1125)	36.7 (413)	1	<0.001	1	<0.001
Yes	13.6 (264)	26.9 (71)	1.08 (0.81 to 1.45)		1.19 (0.87 to 1.61)		23.1 (337)	18.1 (61)	0.38 (0.28 to 0.52)		0.38 (0.28 to 0.52)	
Life satisfaction level												
Medium–very high	68.8 (1340)	26.6 (357)	1	0.104	–	n.a.	75.6 (1106)	31.2 (345)	1	0.085	–	n.a.
Low	31.2 (608)	23.2 (141)	0.83 (0.66 to 1.04)		–		24.4 (357)	36.1 (129)	1.25 (0.97 to 1.60)		–	

Unmet STI testing need, defined as reporting one or more new sex partners and/or multiple CAS partners in the lookback period without testing for STIs during the same period.

*Adjusting for: age; sexual identity; ethnicity; country of residence; employment status; HIV status; PrEP use.

†Cisgender MSM.

‡Including ‘Bisexual’ (S1: n=220; S2: n=179); ‘Other’ (S1: n=44; S2: n=50); ‘Straight’ (S1: n=8; S2: n=14).

§Including ‘White British’ (S1: n=1426; S2: n=1045); ‘White Irish’ (S1: n=75; S2: n=55); ‘White other’ (S1: n=227; S2: n=184).

¶Including ‘Black’ (S1: n=31; S2: n=41); ‘Asian’ (S1: n=101; S2: n=63); ‘Mixed or other’ (S1: n=90; S2: n=75).

**The UK government paid 80% of the salary of those who were unable to work due to COVID-19 restrictions.^15^

aOR, adjusted OR; CAS, condomless anal sex; CI, confidence interval; MSM, men who have sex with men; n.a., not applicable; PrEP, pre-exposure prophylaxis; uOR, unadjusted OR.

After adjustments, all other ethnic groups (vs white ethnicity) had greater unmet STI testing need, although just in P1 (aOR: 1.44 (95% CI 1.05 to 1.98), p=0.023). Those living in UK countries outside England (vs those living in England; aOR: 1.71 (95% CI 1.29 to 2.27), p<0.001) and HIV-positive (vs HIV-negative/unknown) MSM (aOR: 2.06 (95% CI 1.48 to 2.86), p<0.001) also had greater unmet testing need during P1, although these associations were not seen in P2. Bisexually-identifying (vs gay-identifying) MSM were found to have greater unmet testing need during P1 (aOR: 1.64 (95% CI 1.23 to 2.18), p=0.001) and P2 (aOR: 1.42 (95% CI 1.06 to 1.90), p=0.019), whereas PrEP users (vs non-PrEP users) had less unmet testing need during P2 (aOR: 0.38 (95% CI 0.28 to 0.52), p<0.001).

When an interaction term for survey period was included, the likelihood of having unmet STI testing need was significantly greater in P2 versus P1 for those unemployed (aOR: 1.51 (95% CI 1.05 to 2.17), p=0.025) and significantly lower for those reporting living with HIV (aOR: 0.42 (95% CI 0.26 to 0.68), p<0.001) and PrEP users (aOR: 0.36 (95% CI 0.23 to 0.55), p<0.001).

### Unmet HIV testing need

Among all HIV-negative/unknown MSM, 22.9% had unmet HIV testing need during P1, increasing to 31.0% during P2, before declining during P3 (25.1%; p<0.001) (table 4).

**Table 4:** Sociodemographic and health-related factors associated with having unmet HIV testing need during three periods of the first year of the UK’s pandemic response

Lookback period (period number)	March–June/July 2020 (P1)						July–November/December 2020 (P2)						December 2020-March/April 2021 (P3)					
	Sample composition, col % (n)	(Row) % have unmet HIV testing need (n)	uOR (95% CI) have unmet HIV testing need	P value	aOR (95% CI)* have unmet HIV testing need	P value	Sample composition, col % (n)	(Row) % have unmet HIV testing need (n)	uOR (95% CI) have unmet HIV testing need	P value	aOR (95% CI)* have unmet HIV testing need	P value	Sample composition, col % (n)	(Row) % have unmet HIV testing need (n)	uOR (95% CI) have unmet HIV testing need	P value	aOR (95% CI)* have unmet HIV testing need	P value
All†	100.0 (1753)	22.9 (402)	–	–	–	–	100.0 (1308)	31.0 (405)	–		–	–	100.0 (1330)	25.1 (334)	–	–	–	–
Sociodemographic characteristics																		
Age (years)																		
Under 30	26.7 (468)	21.8 (102)	1	0.073	1	0.156	28.9 (378)	36.8 (139)	1	0.014	1	0.099	25.8 (343)	24.5 (84)	1	0.280	1	0.350
30–44	35.5 (623)	26.0 (162)	1.26 (0.95–1.67)		1.24 (0.92–1.65)		37.7 (493)	27.8 (137)	0.66 (0.50–0.88)		0.74 (0.55–1.00)		35.1 (466)	23.0 (107)	0.92 (0.66–1.27)		1.02 (0.73–1.43)	
45 and over	37.8 (662)	20.9 (138)	0.94 (0.71–1.26)		0.97 (0.72–1.30)		33.4 (437)	29.5 (129)	0.72 (0.54–0.97)		0.76 (0.56–1.04)		39.1 (520)	27.3 (142)	1.16 (0.66–1.27)		1.23 (0.89–1.69)	
Sexual identity																		
Gay	85.1 (1492)	21.5 (321)	1	0.001	1	0.001	82.4 (1078)	29.0 (313)	1	0.001	1	0.054	83.4 (1109)	22.6 (251)	1	<0.001	1	<0.001
Bisexual‡	14.9 (261)	31.0 (81)	1.64 (1.23–2.19)		1.65 (1.23–2.22)		17.6 (230)	40.0 (92)	1.63 (1.21–2.19)		1.35 (0.99–1.84)		16.6 (221)	37.6 (83)	2.06 (1.51–2.79)		1.84 (1.34–2.52)	
Ethnicity																		
White§	88.5 (1552)	22.0 (342)	1	0.016	1	0.033	87.2 (1141)	30.3 (346)	1	0.196	1	0.116	90.0 (1197)	25.0 (299)	1	0.737	1	0.670
All other ethnic groups¶	11.5 (201)	30.0 (60)	1.51 (1.09–2.08)		1.45 (1.03–2.04)		12.8 (167)	35.3 (59)	1.26 (0.89–1.77)		1.34 (0.93–1.93)		10.0 (133)	26.3 (35)	1.07 (0.71–1.61)		1.10 (0.72–1.68)	
Country of residence in the UK																		
England	85.5 (1499)	22.0 (330)	1	0.030	1	0.011	83.4 (1091)	29.6 (323)	1	0.018	1	0.063	83.4 (1109)	23.8 (264)	1	0.016	1	0.035
Outside England	14.5 (254)	28.4 (72)	1.40 (1.04–1.89)		1.48 (1.10–2.01)		16.6 (217)	37.8 (82)	1.44 (1.07–1.96)		1.35 (0.98–1.85)		16.6 (221)	31.7 (70)	1.48 (1.08–2.03)		1.41 (1.02–1.95)	
Born in the UK																		
Yes	78.3 (1373)	22.1 (304)	1	0.135	–	n.a.	76.3 (998)	32.0 (319)	1	0.157	–	n.a.	78.5 (1044)	24.8 (259)	1	0.626	–	n.a.
No	21.7 (380)	25.8 (98)	1.22 (0.94–1.59)		–		23.7 (310)	27.7 (86)	0.82 (0.62–1.08)		–		21.5 (286)	26.2 (75)	1.08 (0.80–1.45)		–	
Highest educational qualification																		
Degree or higher	58.9 (1031)	23.5 (242)	1	0.530	1	0.883	58.8 (769)	28.7 (221)	1	0.038	1	0.319	56.2 (748)	24.6 (184)	1	0.624	1	0.678
Below degree	41.2 (721)	22.2 (160)	0.93 (0.74–1.17)		0.98 (0.78–1.24)		41.2 (539)	34.1 (184)	1.29 (1.01–1.63)		1.13 (0.88–1.46)		43.8 (582)	25.8 (150)	1.06 (0.83–1.37)		0.95 (0.73–1.23)	
Employed (*inc*. furlough**)																		
Yes	77.7 (1355)	23.8 (322)	1	0.106	–	n.a.	75.4 (986)	30.2 (298)	1	0.311	–	n.a.	78.0 (1037)	23.9 (248)	1	0.059	–	n.a.
No	22.3 (388)	19.9 (77)	0.79 (0.60–1.05)		–		24.6 (322)	33.2 (107)	1.15 (0.88–1.50)		–		22.0 (293)	29.4 (86)	1.32 (0.99–1.76)		–	
Health-related factors																		
PrEP use (*in the lookback period*)																		
No	84.9 (1481)	22.0 (326)	1	0.033	1	0.031	74.2 (970)	36.4 (353)	1	<0.001	1	<0.001	76.8 (1022)	27.8 (284)	1	<0.001	1	0.001
Yes	15.1 (264)	28.0 (74)	1.38 (1.03–1.85)		1.40 (1.03–1.89)		25.8 (337)	15.4 (52)	0.32 (0.23–0.44)		0.35 (0.25–0.48)		23.2 (308)	16.2 (50)	0.50 (0.36–0.70)		0.55 (0.39–0.77)	
Life satisfaction level																		
Medium–very high	69.0 (1208)	23.8 (288)	1	0.190	1	0.128	75.5 (988)	30.6 (302)	1	0.586	1	0.468	75.2 (998)	23.2 (231)	1	0.006	1	0.012
Low	31.0 (543)	21.0 (114)	0.85 (0.66–1.08)		0.82 (0.64–1.06)		24.5 (320)	32.2 (103)	1.08 (0.82–1.41)		1.11 (0.84–1.47)		24.8 (329)	30.7 (101)	1.47 (1.12–1.94)		1.44 (1.08–1.91)	

Unmet HIV testing need, defined as reporting one or more new sex partners and/or multiple CAS partners in the lookback period without testing for HIV during the same period.

*Adjusting for: age; sexual identity; ethnicity; country of residence; education; PrEP use; life satisfaction.

†Cisgender MSM that report a negative or unknown HIV status.

‡Including ‘Bisexual’ (S1: n=210; S2: n=169; S3: n=171); ‘Other’ (S1: n=43; S2: n=47; S3: n=44); ‘Straight’ (S1: n=8; S2: n=14; S3: n=6).

§Including ‘White British’ (S1: n=1288; S2: n=924; S3: n=1000); ‘White Irish’ (S1: n=64; S2: n=50; S3: n=42); ‘White other’ (S1: n=200; S2: n=167; S3: n=155).

¶Including ‘Black’ (S1: n=24; S2: n=38; S3: n=22); ‘Asian’ (S1: n=98; S2: n=61; S3: n=52); ‘Mixed or other’ (S1: n=79; S2: n=68; S3: n=59).

**The UK government paid 80% of the salary of those who were unable to work due to COVID-19 restrictions.^15^

aOR, adjusted OR; CAS, condomless anal sex; CI, confidence interval; MSM, men who have sex with men; n.a., not applicable; PrEP, pre-exposure prophylaxis; uOR, unadjusted OR.

Bisexually-identifying (vs gay-identifying) MSM had greater unmet HIV testing need during P1 (aOR: 1.65 (95% CI 1.23 to 2.22), p=0.001) and P3 (aOR: 1.84 (95% CI 1.34 to 2.52), p<0.001), as did MSM living in countries outside England (vs those living in England) (P1=aOR: 1.48 (95% CI 1.10 to 2.01), p=0.011; P3=aOR: 1.41 (95% CI 1.02 to 1.95), p=0.035). MSM reporting low life satisfaction (vs medium-very high) levels were more likely to have unmet testing need during P3 (aOR: 1.44 (95% CI 1.08 to 1.91), p=0.012). PrEP users (vs non-PrEP users) were less likely to have unmet HIV testing need during P2 (aOR: 0.35 (95% CI 0.25 to 0.48), p<0.001) and P3 (aOR: 0.55 (95% CI 0.39 to 0.77), p<0.001).

When an interaction term for survey period was included, the likelihood of having unmet HIV testing need was significantly greater in P3 versus P2 for those reporting a low level of life satisfaction (aOR: 1.70 (95% CI 1.17 to 2.47), p=0.005). The likelihood of having unmet HIV testing need was significantly lower in P2 versus P1 and in P3 versus P2 for PrEP users (aOR: 0.23 (95% CI 0.15 to 0.36), p<0.001; aOR: 0.37 (95% CI 0.24 to 0.58), p<0.001, respectively).

## Discussion

Large, community-based surveys of MSM living across the UK show a sizeable portion of participants engaged in STI/HIV risk behaviours during the UK’s first national lockdown. The prevalence of risk behaviours increased as restrictions eased and did not decline when restrictions were reinstated in late 2020. Testing for STIs and HIV mirrored these trends, as did having unmet need for testing. Unmet testing need was more common among bisexually-identifying MSM, UK residents living outside England, MSM reporting a low level of life satisfaction and all other ethnic groups except white, at least in the case of STI testing.

Howarth *et al* 6 found a significant drop in sexual risk behaviours reported by MSM during the UK’s first national lockdown in comparison to prepandemic (eg, CAS in the last 3 months; 36.6% vs 55.7%, respectively),^6 13^ although a sizeable proportion continued to report risk behaviour (eg, new male partners in the last 3 months; 46.8% vs 71.1%, respectively). We have observed that as restrictions eased from July 2020, the proportion of MSM reporting risk behaviours increased significantly such that prevalence was returning to prepandemic levels as restrictions eased during summer 2020.^13^ However, although participants’ sociodemographic profiles are largely comparable, the prepandemic 2017 survey sample may have disproportionately recruited those engaged in risk behaviour, given their recruitment through applications primarily used to meet sex partners, possibly overestimating risk behaviour among MSM.^16^ In contrast, the RiiSH-COVID surveys recruited more broadly so perhaps more indicative of behaviour in the general MSM population. In terms of other evidence, it is worth noting that another UK study reported similarly large increases in sexual risk behaviour among MSM as restrictions were eased in summer 2020,^11^ and a Dutch study observed that in the 2 months after the first Dutch lockdown, the proportion of participants reporting CAS had rebounded to prepandemic levels.^17^

STI testing positivity (excluding chlamydia) among those aged ≥25 years increased between 2019 and 2020,^18^ suggesting ongoing STI transmission. Although national surveillance data only provide insight for those engaging with SHS or community-based chlamydia screening, our survey data suggest that unmet need for testing exceeded the rebound in STI/HIV testing after the first national lockdown. This was also found in a Dutch study where only 39% of participants reported catching-up with missed STI/HIV testing after the Netherlands’ first lockdown.^17^ The inability for SHS to meet testing need in the UK could also be due to changes in healthcare seeking behaviour as social restrictions fluctuated.

It is concerning that unmet need—particularly during the first lockdown—appeared to be disproportionately greater among bisexually-identifying MSM, those reporting a low level of life satisfaction and all other ethnic groups except white, who already experience disproportionate STI/HIV burden.^13 19 20^ Previous research has linked low psychological well-being with greater participation in risk behaviour.^21^ We found such an association during P3, with participants reporting poorer mental health more likely to have unmet HIV testing need, but no more likely to report HIV testing; this potentially reflects an impact on engaging in sexual risk behaviour after experiencing ‘lockdown fatigue’ and declining mental health resilience.^22^

We used the same study protocol and similar recruitment methods for each RiiSH-COVID survey and an earlier survey undertaken in 2017, resulting in large samples with broadly comparable sociodemographic profiles, and enabling comparisons with a prepandemic ‘benchmark’.^12 13^ Our findings also complement national surveillance data on SHS attendees by providing community-recruited samples of MSM, thereby enabling comparisons on risk behaviours and testing need in MSM who do and do not access SHS.

However, there are limitations. As cross-sectional surveys, associations between variables can be bidirectional and therefore we cannot infer causality. We also acknowledge the issue of temporality in participants’ behaviours. Additionally, the lookback periods in each round were of unequal length, varying by a few weeks, and thus providing participants in different rounds with greater (or lesser) opportunity to engage in risk behaviour and/or use services. This reflects the pragmatic nature of the surveys and the decision to prioritise lockdown ‘milestones’ (eg, the start of a full lockdown) rather than specific dates, which would be more burdensome for participants to recall, and because of differences between the UK’s four nations in the exact date of these changes. However, the results from other studies suggest differences in lookback periods are unlikely to explain away our findings.^11 17^ Recruitment through an online survey and through social media and dating applications will exclude MSM who do not use these platforms, are not seeking new sex partners and/or do not have internet access; potentially limiting the generalisability of our findings to all MSM. STI testing data were not available for P3 thereby limiting some comparisons. Nevertheless, guidelines state the importance of STI testing alongside HIV testing such that trends observed for HIV testing and unmet need during P3 may well reflect those for STIs.^1 23^

Given the small number of migrants and participants from ethnic minority groups (despite attempts to boost the number of participants from these groups by using different images and social media platforms to promote our survey), we needed to categorise country of birth and ethnicity as binary variables thereby overlooking substantive differences in sexual health within these groups.^13 19^ Likewise, as the majority of participants were cisgender MSM, we were unable to make meaningful inferences on barriers to access and sexual health needs of gender minorities.^24 25^ The variables we derived to try and capture unmet STI/HIV testing need were informed by national guidelines,^1^ which advise quarterly STI and HIV testing in MSM engaging in certain risk behaviours. We acknowledge that this is a crude measure and does not take account of subjective risk, for example, participants needing to test for HIV if their partner has an undetectable HIV viral load,^26^ but we were unable to measure this in our surveys.

The high proportion of MSM considered to have unmet STI/HIV testing need throughout the periods of social restrictions and reconfiguration of SHS provision should be of concern to sexual healthcare workers and policymakers, particularly its disproportionate impact on certain groups. There are many reasons why MSM may have been less likely to test and/or have postponed testing, including: considering it a lower priority in the context of the ongoing pandemic; wanting to avoid perceived potential for judgement by SHS; avoid potential COVID-19 transmission from attending SHS in-person; or being unable to adjust to SHS reconfiguration to remote services. Our observations suggest that for some men, such as those taking HIV PrEP (where we observed an almost doubling of reported use between P1 and P2 likely due to the introduction of routinely commissioned and available PrEP in the latter half of 2020^27^), SHS were able to meet their testing needs. In contrast, SHS may need to target testing campaigns and provision to those with relatively high unmet need, including bisexually-identifying MSM, ethnic minorities and those whose mental health has been most adversely affected by the pandemic. Ensuring flexible, equitable access to SHS is essential in meeting need and widening access to testing, and offers opportunities for proactive promotion of HIV PrEP services to those eligible.

There is evidence that sexual risk behaviour in MSM rebounded to prepandemic levels once restrictions were eased.^11 17^ Given the ongoing COVID-19 pandemic, and with many countries implementing new restrictions towards the end of 2021,^28^ there is a need for increased testing capacity in SHS and targeted testing campaigns to accommodate testing backlogs and to meet need related to rebounding risk behaviours and STI/HIV transmission as restrictions ease. To mitigate inequalities in access, ongoing surveillance and observational research can help identify whether newly reconfigured remote services are equitable and, indeed, whether, where and for whom in-person services must remain open.

## Data Availability

Data are available on reasonable request.
