# Supplementary material for: Changes in STI and HIV testing and testing need among men who have sex with men during the UK’s COVID-19 pandemic response
Source: Sex Transm Infect. 2022 Jul 21;99(4):226–38. doi: 10.1136/sextrans-2022-055429 (PMC10313956; doi:10.1136/sextrans-2022-055429)
Supplement: Supplementary data [file sextrans-2022-055429supp001.pdf]

Appendix 1 Timeline of RiiSH-COVID surveys in relation to COVID-19 social restrictions during the first year of the UK's pandemic response

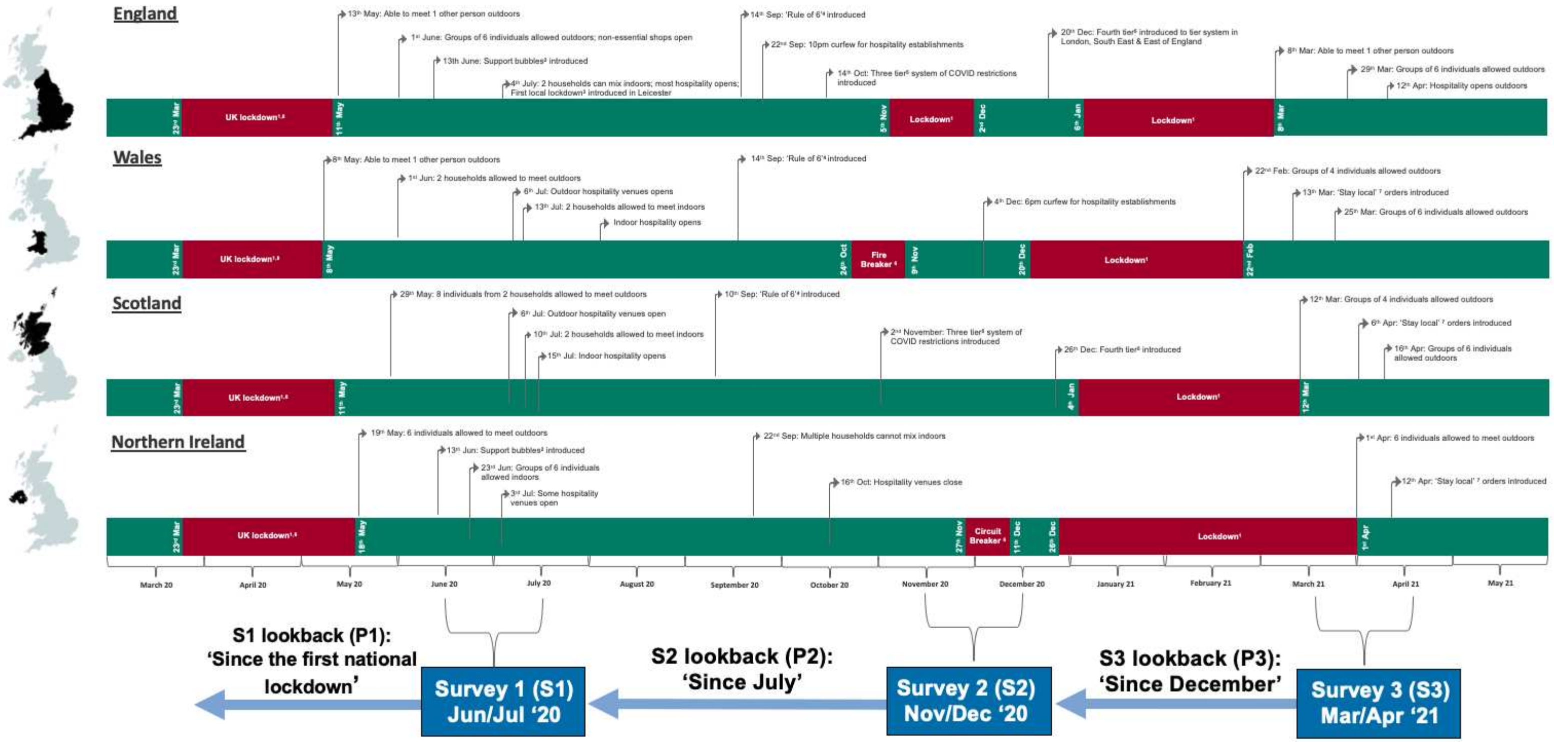

1 'Stay at home' orders implemented across the country, these include: Only leaving home for food, medical reasons, exercise or work; Working from home unless you are unable to do so; Essential shops can remain open.
2 'Support bubbles': Individuals living alone, households with one adult, households with children under the age of one, households with children who have a disability, aged 16 or 17 living without an adult, can join no more than one other household to form a 'bubble' under which they are essentially classed as one household.
3 'Local lockdown': stay at home orders¹ continue to be in form in some local authorities across the UK. Individuals are discouraged from travelling from/to these areas.
4 'Rule of 6': Apart from in work and school environments, any social gatherings of more than 6 individuals is against the law
5 'Tier system': Differing levels of social restrictions, categorised into tiers 1-4 are introduced and can be implemented at local authority levels. Descriptions of tiers can be found here: https://www.gov.uk/government/publications/tier-posters-medium-high-and-very-high
6 'Fire/circuit breaker': a short period of stay at home (lockdown) orders to limit rising infections
7 'Stay local': Restrictions on non-essential travel across local authority boundaries remains in place, individuals encouraged to stay within their local council boundaries for non-essential shopping etc.
8 'Social distancing': Across the UK, individuals were encouraged to keep a 2 meter distance from other people. This was later adapted to 1 meter plus, which included keeping a 1 meter distance from other people in addition to taking another precaution, these included: wearing a face covering; separated by plastic screens; being outdoors.

Appendix 3 Sociodemographic and health-related characteristics reported by participants in the RiiSH-COVID surveys conducted during three periods of the first year of the UK's pandemic response

Survey<br>Participants' characteristics ¶	RiiSH-COVID survey 1 (S1)<br>Column % (n)	RiiSH-COVID survey 2 (S2)<br>Column % (n)	RiiSH-COVID survey 3 (S3)<br>Column % (n)	**p-value** *(overall difference across surveys)*
**Socio-demographics**				
**Age** *(years)*	N=1950	N=1463	N=1486	
Under 30	24.6 (479)	26.5 (388)	23.8 (353)	0.040
30-44	35.3 (688)	37.8 (553)	35.5 (528)	
45 and over	40.2 (783)	35.7 (522)	40.7 (605)	
**Sexual identity**	N=1950	N=1463	N=1487	
Gay	86.1 (1678)	83.4 (1220)	84.2 (1252)	0.083
Bisexual §	14.0 (272)	16.6 (243)	15.8 (235)	
**Ethnicity**	N=1950	N=1463	N=1487	
White	88.6 (1728)	87.8 (1284)	90.0 (1338)	0.058
Black	1.6 (31)	2.8 (41)	1.8 (27)	
Asian	5.2 (101)	4.3 (63)	3.6 (54)	
Mixed or other	4.6 (90)	5.1 (75)	4.6 (68)	
**Country of residence in the UK**	N=1950	N=1463	N=1487	
England	86.1 (1679)	84.3 (1233)	83.8 (1246)	0.097
Scotland	7.4 (144)	9.8 (144)	9.4 (139)	
Wales	4.2 (82)	4.3 (63)	4.9 (73)	
Northern Ireland	2.3 (45)	1.6 (23)	2.0 (29)	
**Born in the UK**	N=1950	N=1463	N=1487	
Yes	78.1 (1523)	76.9 (1125)	78.8 (1171)	0.467
**Highest educational qualification**	N=1949	N=1463	N=1487	
Degree or higher	59.0 (1149)	57.4 (840)	55.6 (827)	0.146
**Employed** *(inc. furlough*)*	N=1939	N=1463	N=1487	
Yes	77.2 (1497)	74.9 (1095)	77.6 (1154)	0.154
**Living with partner(s)**	N=1950	N=1463	N=1487	
Yes	31.0 (604)	29.5 (432)	32.8 (488)	0.153

**Health related factors**				
**HIV status**	N=1950	N=1463	N=1487	
Positive	10.1 (197)	10.6 (155)	10.6 (157)	0.868
**Currently on anti-retroviral therapy for HIV**	N=197	N=155	N=157	
Yes	95.9 (189)	98.1 (152)	97.5 (153)	0.473
**Undetectable viral load** *(last time checked)*	N=197	N=155	N=157	
Yes	94.9 (187)	97.4 (151)	96.2 (151)	0.840
**PrEP use** *(in the lookback period)*	N=1941	N=1462	N=1487	
Yes	13.6 (264)	23.1 (337)	20.7 (308)	<0.001
**How user took PrEP** *(in the lookback period)*	N=264	N=337	N=308	
Daily	35.6 (94)	50.7 (171)	55.8 (172)	<0.001
4-6 times a week	8.3 (22)	8.9 (30)	10.4 (32)	
Intermittently (e.g. week on/week off)	12.9 (34)	5.0 (17)	3.9 (12)	
Before and after sex (event based/on demand)	28.4 (75)	29.1 (98)	20.5 (63)	
One-off(s)	7.6 (20)	4.2 (14)	4.9 (15)	
Other	7.2 (19)	2.1 (7)	4.6 (14)	
**Life satisfaction level †**	N=1948	N=1463	N=1484	
Low	31.2 (608)	24.4 (357)	24.7 (367)	<0.001
**Anxiety level †**	N=1943	N=1460	N=1484	
Low/very low	54.0 (1050)	41.2 (601)	44.8 (665)	<0.001

¶ Cis-gender MSM

§ including ‘Bisexual’ (S1: n=220; S2: n=179; S3: n=180); ‘Other’ (S1: n=44; S2: n=50; S3: n=47); ‘Straight’ (S1: n=8; S2: n=14; S3: n=8)

† personal well-being (using the Office for National Statistics (ONS) well-being measures); Descriptions/definitions available at: https://www.ons.gov.uk/peoplepopulationandcommunity/wellbeing/methodologies/surveysusingthe4officefornationalstatisticspersonalwellbeingquestions

* the UK government paid 80% of the salary of those who were unable to work due to COVID-19 restrictions [15]

Appendix 4 Sexual behaviour reported by participants in the RiiSH-COVID surveys conducted during three periods of the first year of the UK's pandemic response

**Lookback period (period number)**	**March-June/July 2020 (P1)**	**July-November/December 2020 (P2)**	**December 2020-March/April 2021 (P3)**	**p-value** *(overall difference across periods)*
	**Column % (n)**	**Column % (n)**	**Column % (n)**	
**Sexual behaviours** *(in the lookback period)* ¶				
**Sex with a man**	N=1950	N=1463	N=1487	
None	22.8 (445)	10.5 (153)	14.3 (212)	<0.001
Virtual only	14.4 (280)	6.1 (89)	8.7 (129)	
Physical	62.8 (1225)	83.5 (1221)	77.1 (1146)	
**No. of new physical sex* partners**	N=1949	N=1460	N=1483	
None	63.0 (1227)	38.3 (559)	48.2 (715)	<0.001
One	13.6 (264)	14.4 (210)	14.5 (215)	
Two or more	23.5 (458)	47.3 (691)	37.3 (553)	
**≥5 new physical sex partners**	N=1949	N=1460	N=1483	
Yes	8.1 (158)	21.8 (318)	14.4 (214)	<0.001
**No. of CAS partners**	N=1950	N=1463	N=1487	
None	62.1 (1211)	43.2 (632)	48.5 (721)	<0.001
One	20.4 (398)	23.7 (346)	23.9 (356)	
Two or more	17.5 (341)	33.2 (485)	27.6 (410)	
**Chemsex**	N=1950	N=1463	N=1487	
Yes	3.7 (72)	6.0 (87)	3.8 (56)	0.002

¶ Cis-gender MSM

* Physical sex, defined as: any activity intended to achieve orgasm (or close to) for one or both partners

Appendix 5 Sexual health service use reported by participants in the RiiSH-COVID surveys conducted during three periods of the first year of the UK's pandemic response

**Lookback period (period number)**	**March-June/July 2020 (P1)**	**July-November/December 2020 (P2)**	**December 2020-March/April 2021 (P3)**	**p-value** *(overall difference across periods)*
	**Column % (n)**	**Column % (n)**	**Column % (n)**	
**HIV-testing ¶**				
**Ever tested for HIV**	N=1950	N=1463	N=1487	
Yes	87.5 (1707)	89.7 (1313)	88.5 (1316)	0.390
**Where tested** if tested in the lookback period	N=521	N=515	N=538	
In-person	26.1 (136)	53.8 (277)	44.6 (240)	<0.001
Self-sample	73.7 (384)	46.2 (238)	55.4 (538)	
**Tried (and failed) to get a HIV test** *in the lookback period*	N=989	N=643	N=621	
Yes	11.6 (115)	11.4 (73)	18.8 (117)	<0.001
**Confident accessing a HIV test** *if not tested in the lookback period*	N=1232	N=793	N=792	
Yes	60.4 (744)	65.6 (520)	73.7 (584)	<0.001
**STI testing ¶**				
**Ever tested for STIs**	N=1950	N=1463		
Yes	81.7 (1593)	83.0 (1214)	-	0.330
**Results** *of most recent STI test*	N=487	N=544		
Positive for at least 1	17.3 (84)	22.6 (123)	-	0.096
**Where tested** *if tested in the lookback*	N=487	N=544		
In-person	34.5 (168)	60.9 (331)	-	<0.001
Self-sample	65.3 (318)	39.0 (212)	-	
**Tried (and failed) to get an STI test** *in the lookback*	N=1950	N=1463		
Yes	7.6 (148)	6.2 (90)	-	0.103
**Confident accessing STI testing** *if not tested in*	N=1106	N=669		

the lookback				
Yes	60.8 (672)	66.4 (444)	-	0.018

¶ Cis-gender MSM
