# Supplementary material for: Changes in STI and HIV testing and testing need among men who have sex with men during the UK’s COVID-19 pandemic response
Source: Sex Transm Infect. 2022 Jul 21;99(4):226–38. doi: 10.1136/sextrans-2022-055429 (PMC10313956; doi:10.1136/sextrans-2022-055429)
Supplement: Supplementary data [file sextrans-2022-055429supp004.pdf]

# Your sex life since lockdown

Welcome to *Your sex life since lockdown.* This is a voluntary survey about sexually transmitted infections (STIs), use of sexual health clinics and sexual behaviour before and during the COVID-19 pandemic.

### Please take part if you

- Are a man OR a trans man OR a trans woman OR a non-binary person assigned male at birth

- AND you have sex with men and/or other people assigned male at birth

- AND are aged 16 years or over

- AND you live in the UK

This is the third time we are asking people to complete the survey. You can take part whether you did or you didn't complete the first and/or second surveys in 2020.

### What is the survey about?

It asks about your thoughts about sexually transmitted infections (STIs), your sexual behaviour, and your use of health services including sexual health clinics before, during and after the first national lockdown on 23 March 2020 when social distancing measures were first introduced by the UK government due to the COVID-19 pandemic. This information will be used to help plan better sexual health services.

### How long does the survey take to do?

Most people take about 10 minutes to complete the survey.

### What about data protection?

This survey is completely anonymous. We will NOT collect any information that identifies the device you are using (phone or computer) or try to install any cookies on it. We will not collect any information about you that would allow anybody to identify you. A report of the results will be produced at the end of the study. It will be available from our study website. No one will be identifiable from the report.

### Who are we?

The National Institute for Health Research (NIHR) Health Protection Research Unit in Blood Borne and Sexually Transmitted Infections is led by University College London in partnership with Public Health England. The unit aims to conduct state-of-the-art research to improve the health of the population, and to help develop practical policy guidelines for those working in health protection. If you have any questions or concerns about the study, please contact David Reid via email (david.reid@ucl.ac.uk).

Please read the statements below carefully and tick the appropriate box to indicate whether you agree to participate in this survey

- I have read and understood the above information and I agree to participate in the survey
- I do not want to take part in this survey

Are you aged 16 years or over?

- I am aged 16 or over
- I am aged under 16

Are you currently living in the UK?

- Yes
- No

## About you

How old are you?

- 16
- 17
- etc 80
- Over 80

Which of the following options best describes how you think of yourself?

- Man (including trans man)
- Woman (including trans woman)
- Non-binary
- In another way

What sex were you assigned at birth?

- Male
- Female
- Intersex
- Prefer not to say

Since the beginning of last year (2020), have you had sex with... [*tick all that apply*]

- Men (including trans men)
- Women (including trans women)
- Non-binary people assigned male at birth
- Non-binary people assigned female at birth
- People who identify in another way
- No one

*Thank you for your interest but your answers show that you do not qualify to take part in this survey. Please press submit on the next page to exit and you will see some links to information on sexual health and support services.*

## About you

How would you describe your sexual orientation?

- Straight or heterosexual
- Gay or homosexual
- Bisexual
- I don't use any terms
- Other
- Rather not say

Please describe your sexual orientation:

Which of the following best describes your ethnic group?

- White British
- White Irish
- Any other White background
- Black or Black British: Caribbean
- Black or Black British: African
- Black or Black British: Any other Black background
- Asian/Asian British: Indian
- Asian/Asian British: Pakistani

- Asian/Asian British: Bangladeshi
- Asian/Asian British: Chinese
- Asian/Asian British: Any other Asian background
- Mixed: White and Black Caribbean
- Mixed: White and Black African
- Mixed: White and Asian
- Any other mixed/multiple background
- Any other ethnic group

Please describe what other white background?

Please describe what other black background?

Please describe what other Asian background?

Please describe what other mixed/multiple background?

Please describe what other ethnic group?

Have you used any of the following social networking and dating apps in the past 3 months? [*tick all that apply*]

- Facebook
- Grindr
- Instagram
- Twitter
- None of these

## COVID-19

In the following questions, we ask about your life and activities before, during and after the '***first national lockdown***' which is when social distancing measures were first introduced on 23 March 2020 by the UK government, due to the COVID-19 (coronavirus) pandemic.



Brown JRG, *et al. Sex Transm Infect* 2023; 99:1–13. doi: 10.1136/sextrans-2022-055429

In this questionnaire, we focus on your life and activities in the last four months, since ***December 2020***.

This survey is completely anonymous. We do NOT collect any information that identifies you or the device you are using.

## Where you live and where you were born

Were you born in the UK?

- No
- Yes

How many years have you been living in the UK?

- Less than 1 year
- 1
- etc
- More than 80

Which part of the UK are you currently living in?

- England
- Scotland
- Wales
- Northern Ireland

Which local authority are you currently living in?

- I don't know
- Barking etc
- York

Which council area are you currently living in?

- I don't know
- Aberdeenshire
- Angus etc
- West Lothian

Which council area are you currently living in?

- I don't know
- Antrim and Newtownabbey Borough etc

- Newry, Mourne and Down District

Which local authority are you currently living in?

- I don't know
- Blaenau Gwent Bridgend etc
- Wrexham

Is this your usual place of residence?

- No
- Yes

Did you move from your usual place of residence because of the first national lockdown?

- No
- Yes

## About your education and employment

What is your highest educational qualification?

- I have no educational qualifications
- GCSEs/O-Levels/National 5 or equivalent
- A-levels/Scottish Highers or equivalent
- Higher education below degree level (e.g. HNC, HND)
- Degree or higher
- Other

Which of the following best describes your current situation?

- On furlough
- Employed on reduced hours (due to COVID / lockdown) AND on furlough
- Employed on reduced hours (due to COVID / lockdown) AND NOT on furlough
- Job made redundant (due to COVID / lockdown)
- Employed full-time
- Employed part-time
- Self-employed
- No longer self-employed (due to COVID / lockdown)

- Self-employed on reduced hours (due to COVID / lockdown)
- Unemployed
- Full-time student
- Part-time student
- Retired
- Long-term sick leave / medically retired
- Other

{if more than one response to above question, only response options ticked are included here} Which of **one** the following best describes your current situation?

## About your household

Who are you living with currently? [*tick all that apply*]

- I live alone
- A partner / partners
- My child / children
- Other family member(s)
- Friend(s) / housemate(s)
- Other(s)

## HIV testing

Have you **ever** received an HIV test result?

- No, I've never received an HIV test result
- Yes, I have tested positive (I'm living with HIV)
- Yes, my last test was negative (I did not have HIV at the time of the test)

When did you **last** have an HIV test?

- Since December 2020
- Between 23 March 2020 (first national lockdown) and December 2020
- Before 23 March 2020 (first national lockdown)

Where did you have your **last** HIV test?

- At a sexual health clinic
- At another type of hospital (outpatients) clinic
- At my GP practice
- At a private medical practice
- At a hospital as an inpatient (staying overnight)
- At a community HIV testing service (that is not in a hospital or clinic)
- At a blood donation service
- I used a free online self-sampling service [I took my own sample
- and sent it off for the result]
- I used a private online self-sampling service (I had to pay for it) [I took my own sample and sent it off for the result]
- I used a self-testing kit [I took my own sample and found out the result immediately]
- At a mobile medical unit
- Somewhere else

Have you tried to get an HIV test since **December 2020**?

- No
- Yes

Where have you tried to get an HIV test since **December 2020**? [*tick all that apply*]

- At a sexual health clinic
- At another type of hospital (outpatients) clinic
- At my GP practice
- At a private medical practice
- At a hospital as an inpatient (staying overnight)
- At a community HIV testing service (that is not in a hospital or clinic)
- At a blood donation service
- Using a free online self-sampling service [taking my own sample and sending it off for the result]
- Using a private online self-sampling service (paying for it) [taking my own sample and sending it off for the result]
- Using a self-testing kit [taking my own sample and finding out the result immediately]

- At a mobile medical unit
- Somewhere else

How confident are you that you could have got an HIV test since **December 2020** if you wanted one?

- Very confident
- Quite confident
- A little confident
- Not at all confident
- I don't know

## Living with HIV

In which year were you first diagnosed with HIV?

- 2021
- etc 1984
- Before 1984

Were you first diagnosed with HIV in the last 12 months?

- No
- Yes

Are you currently taking antiretroviral treatment (sometimes known as ART or HAART)?

- No
- Yes

What was the result of your viral load test the **last** time you had it checked?

- Undetectable
- Detectable
- I was told but I don't remember the result
- It was measured but I was not told the result
- It was not measured
- I don't remember

## Use of PrEP

*PrEP is a drug taken by HIV-negative people that reduces the risk of getting HIV.*

Q39, Have you **ever** used PrEP (including if you are currently using PrEP)?

- No
- Yes
- I don’t know

Q41 When did you **first** take PrEP? [*Please estimate if you can’t say exactly*]

Month

- January
- February
- March
- April
- May
- June
- July
- August
- September
- October
- November
- December

Q 42Year

- Before 2017
- 2017
- 2018
- 2019
- 2020
- 2021

When did you **last** take PrEP? [*Please estimate if you can’t say exactly*]

Q43Month

- January
- February
- March
- April
- May
- June
- July
- August
- September
- October
- November
- December

Q44 Year

- Before 2017
- 2017
- 2018
- 2019
- 2020
- 2021

Did you **first** take PrEP before or after the first national lockdown (23 March 2020)?

- After first national lockdown
- Before first national lockdown

Did you **last** take PrEP before or after the first national lockdown (23 March 2020)?

- After first national lockdown
- Before first national lockdown

Thinking about the time since **December 2020**, how often have you taken PrEP: [*tick all that apply*]

- Daily
- 4-6 times a week
- Intermittently (e.g. a week on a week off)

- Before and after sex (event based or on demand)
- One-off(s)
- Other

Thinking about the time **between 23 March 2020 (first national lockdown)** and **December 2020**, how often were you taking PrEP: [*tick all that apply*]

- I didn't use PrEP during this time
- Daily
- 4-6 times a week
- Intermittently (e.g. a week on a week off)
- Before and after sex (event based or on demand)
- One-off(s)
- Other

## Sexually transmitted infection (STI) testing (other than HIV)

Have you **ever** had a test for a sexually transmitted infection (STI) other than HIV?

- No
- Yes

When did you **last** have a test for a sexually transmitted infection (STI) other than HIV?

- Since December 2020
- Between 23 March 2020 (first national lockdown) and December 2020
- Before 23 March 2020 (first national lockdown)

Have you received a test result for a sexually transmitted infection (STI) other than HIV since **December 2020**?

- No, I haven't received any STI results yet
- Yes, all my STI test results were negative
- Yes, I tested positive for at least one STI

Where have you tested for STIs other than HIV since

**December 2020**? *[tick all that apply]*

- At a sexual health clinic
- At an HIV clinic
- At my GP practice
- At a private medical practice
- At a community HIV testing service (that is not in a hospital or clinic)
- I used a free online self-sampling service [I took my own sample and sent it off for the result]
- I used a private online self-sampling service (I had to pay for it) [I took my own sample and sent it off for the result]
- At a mobile medical unit
- Somewhere else

Have you tried to get a test for a sexually transmitted infection (STI) other than HIV since **December 2020**?

- No
- Yes

Where have you tried to get a test for a sexually transmitted infection (STI) other than HIV since **December 2020**? [*tick all that apply*]

- At a sexual health clinic
- At an HIV clinic
- At my GP practice
- At a private medical practice
- At a community HIV testing service (that is not in a hospital or clinic)
- Using a free online self-sampling service [taking my own sample and sending it off for the result]
- Using a private online self-sampling service (paying for it) [taking my own sample and sending it off for the result]
- At a mobile medical unit
- Somewhere else

How confident are you that you could have got an STI test since **December 2020** if you wanted one?

- Very confident

- Quite confident
- A little confident
- Not at all confident
- I don't know

## Sexually transmitted infection (STI) testing (other than HIV)

*We are aware that there are lots of reasons why people do or do not get tested for sexually transmitted infections (STIs) other than HIV. We are interested in hearing your views on this.*

*The following statements are about testing for **sexually transmitted infections (STIs) other than HIV**. (e.g. Chlamydia, Gonorrhea, Syphilis), either at a sexual health clinic or at home by ordering a test online or by phone, taking your own samples and sending them off by post for the test result. Please think about how you feel at this moment when indicating the extent to which you **disagree or agree** with each statement.*

- Strongly disagree
- Disagree
- Neither disagree nor agree
- Agree
- Strongly agree

I only think about having an STI test if I have symptoms.

I know how to get an STI test at the moment.

I know what the current recommendations are for when and how often I should get tested.

I would feel confident to collect my own finger prick blood sample.

I would feel confident to collect my own urine sample.

I would feel confident to collect my own swab sample. (e.g. throat/anal/vaginal swab).

Arranging to have an STI test is difficult for me.

Taking my own STI test samples at home is convenient.

It is convenient to get an STI test at a clinic.

I think my partners would want me to be tested for STIs.

People I know have STI tests.

I worry that staff at a sexual health clinic would judge me for seeking an STI test.

Getting tested for STIs regularly protects me and my partner/s.

I intend to have an STI test in the next 3 months.

I believe I am at risk of getting an STI at the moment.

I think that STI samples taken at home and sent back for results are accurate.

I am afraid of STI test results.

I would feel ashamed if I had an STI.

I would feel embarrassed to be seen in a sexual health clinic.

Having a regular STI test has become a habit.

## Vaccination

***Hepatitis A*** *is an infection of the liver caused by a virus that's spread in the poo of an infected person. Occasionally this could happen during sex.*

Have you ever received the **hepatitis A** vaccine?

- No
- Yes
- I don't know

*Hepatitis A vaccination usually involves two doses, given by injection, between 6 and 12 months apart.*

When did you **last** receive the hepatitis A vaccine (your most recent injection)?

- Since December 2020
- Before December 2020 but can't remember when
- Between 23 March 2020 (first national lockdown) and December 2020

- Before 23 March 2020 (first national lockdown)

***Hepatitis B** is an infection of the liver caused by a virus that's spread through blood and body fluids.*

Have you ever received the **hepatitis B** vaccine?

- No
- Yes
- I don't know

*Hepatitis B vaccination involves having three doses, given by injection, at the recommended intervals. These intervals can vary between a few weeks to several months.*

When did you **last** receive the hepatitis B vaccine (your most recent injection)?

- Since December 2020
- Before December 2020 but can't remember when
- Between 23 March 2020 (first national lockdown) and December 2020
- Before 23 March 2020 (first national lockdown)

***HPV** (Human papillomavirus) is the name of a very common group of viruses. They do not cause any problems in most people, but some types can cause genital warts or cancer.*

*Have you ever received the **HPV** vaccine?*

- No
- Yes
- I don't know

*HPV vaccination involves having three doses, given by injection, at the recommended intervals. If you receive the vaccine before the age of 15 you only need two doses.*

When did you **last** receive the HPV vaccine (your most recent injection)?

- Since December 2020
- Before December 2020 but can't remember when
- Between 23 March 2020 (first national lockdown) and December 2020

- Before 23 March 2020 (first national lockdown)

## About sexual health symptoms

Have you had any of the following symptoms since **December 2020**? [*tick all that apply*]

- I haven't had any symptoms
- Pain, burning or stinging when passing urine
- Passing urine more often than usual
- Genital wart or lump
- Genital ulcer or sore
- Abnormal vaginal discharge
- Vaginal pain during sex
- Pain during sex
- Abnormal bleeding between periods
- Bleeding after sex (not during a period)
- Lower abdominal or pelvic pain (not related to periods)
- Pain, bleeding or discharge from the rectum
- Discharge from the end of the penis
- Painful testicles

Have you seen a healthcare professional about your symptoms since **December 2020** [*please include telephone and video/online consultations, not just face-to- face*]?

- Yes – I have seen a healthcare professional
- No – I have not seen a healthcare professional
- No – but I have an appointment

What type of appointment(s) did you have? [*tick all that apply*]

- Face-to-face
- Telephone
- Video / online

What type of appointment have you booked?

- Face-to-face
- Telephone

- Video / online

## About sexual health clinics

### Have you **ever** visited a sexual health clinic?

- No
- Yes

### When was the **last** time you visited a sexual health clinic?

- Since December 2020
- Between 23 March 2020 (first national lockdown) and December 2020
- Before 23 March 2020 (first national lockdown)

### Why did you attend a sexual health clinic (the **last** time if more than once)? [*tick all that apply*]

- I wanted a general sexual health check-up
- I had no symptoms, but I was worried I might have an STI or HIV
- I had symptoms
- A sexual partner had symptoms
- A sexual partner was diagnosed with an STI
- Treatment after a previous positive test
- Check-up after a previous positive test
- As follow-up to an online test
- Ongoing HIV care and treatment
- To get PEP to prevent HIV
- To get PrEP to prevent HIV
- Following sexual assault
- Following domestic violence
- I was told to attend by my GP/family doctor or another healthcare professional
- I needed condoms
- I needed contraception (other than condoms)
- I needed a vaccination
- I couldn’t get an online testing kit
- For another reason

## Your sexual relationships

*In this survey we use the term 'steady relationships' to refer to relationships with, for example, boyfriends, girlfriends, husbands or wives which mean that you are NOT 'single'. Do not include relationships with partners who are purely sex buddies.*

*We use ‘men’ to include ‘trans men’ and we use ‘women’ to include ‘trans women’.*

Are you currently in a steady relationship? [*tick all that apply*]

- Yes, with a man (including trans men)
- Yes, with more than one man (including trans men)
- Yes, with a woman (including trans women)
- Yes, with more than one woman (including trans women)
- Yes, with a person who identifies in another way
- Yes, with more than one person who identifies in another way
- No, I'm single

For how many years have you been in your steady relationship with a man? [*if you have more than one steady relationship with a man, please tell us about the longest*]

- Less than 1 year
- 1
- 2
- etc
- 25
- More than 25 years

Do you and this steady male partner have the same HIV status?

- Yes, we have the same HIV status (both positive or both negative)
- No, one of us is positive and the other is negative
- I don’t know whether we have the same status or not

Are you and this steady male partner currently living together in the same household?

- No

- Yes

For how many years have you been in your steady relationship with a woman? [*if you have more than one steady relationship with a woman, please tell us about the longest*]

- Less than 1 year
- 1
- 2 etc
- 25
- More than 25 years

Do you and this steady female partner have the same HIV status?

- Yes, we have the same HIV status (both positive or both negative)
- No, one of us is positive and the other is negative
- I don’t know whether we have the same status or not

Are you and this steady female partner currently living together in the same household?

- No
- Yes

For how many years have you been in your steady relationship with someone who identifies in another way? [*if you have more than one steady relationship with a person who identifies in another way, please tell us about the longest*]

- Less than 1 year
- 1 etc
- 24
- 25
- More than 25 years

Do you and this steady partner have the same HIV status?

- Yes, we have the same HIV status (both positive or both negative)
- No, one of us is positive and the other is negative
- I don’t know whether we have the same status or not

Are you and this steady partner currently living together in the same household?

- No
- Yes

## About sex with men since December 2020

*In the following questions, we ask about sex since December 2020. Please say when you last did something EVEN if this was not typical for you. This survey is anonymous and confidential.*

Q84 Thinking of the time since December 2020, have you had any kind of sex with a man (any activity intended to achieve orgasm (or close to orgasm) for one or both partners)?

- No
- Yes – virtual sex (e.g. phone sex, sexting)
- Yes – sex with physical contact (you could touch each other)
- Yes – virtual sex and sex with physical contact

Q85. Thinking of the time since December 2020, how many men have you had any kind of **physical sexual contact** with during this time [*please include any sexual contact, not just anal intercourse*]?

- 1
- 2
- 3
- 4
- 5
- 6
- 7
- 8
- 9
- 10
- 11-20
- 21-30
- 31-40
- 41-50
- More than 50



Brown JRG, *et al. Sex Transm Infect* 2023; 99:1–13. doi: 10.1136/sextrans-2022-055429

Is this man a new sex partner (you had sex with him for the first time since December 2020)?

- No
- Yes

How many of these {number} men are new partners who you had sex with for the first time since December 2020?

- None
- 1
- 2
- 3
- 4
- 5
- 6
- 7
- 8
- 9
- 10
- 11-20
- 21-30
- 31-40
- 41-50
- More than 50

## Meeting male sexual partners since December 2020

### Q88 In which of the following ways have you met or made contact with male partners for physical sexual contact since December 2020? [*tick all that apply*]

- A website for gay or bisexual men
- A smartphone app that locates potential partners and tells you how far away they are
- A gay sex party in a private home
- A gay community centre, gay organisation or gay social group (physical not virtual venues)

- A cruising location (street, roadside service area, park, beach, lavatory)
- Through friends
- In my household
- In a support bubble with another household
- Elsewhere

## About anal sex with men since December 2020

*We use the term "anal sex" to mean sex where one partner puts their penis into the other partner's anus, whether or not this ends in ejaculation.*

Thinking of the time since December 2020, have you had anal sex with a man (either with or without a condom)?

- No
- Yes

Since December 2020, have you had condomless anal sex with a man?

- No
- Yes

Since December 2020, how many men have you had condomless anal sex with?

- 1
- 2
- 3
- 4
- 5
- 6
- 7
- 8
- 9
- 10
- 11-20
- 21-30

- 31-40
- 41-50
- More than 50

Do you and this man have the same HIV status?

- Yes, we have the same HIV status (both positive or both negative)
- No, one of us is positive and the other is negative
- I don’t know whether we have the same status or not

How many of these {number} men you had condomless anal sex with did you know had the same HIV status as yourself (both positive or both negative)?

- I don’t know
- None
- 1
- 2
- 3
- 4
- 5
- 6
- 7
- 8
- 9
- 10
- 11-20
- 21-30
- 31-40
- 41-50
- More than 50

## About anal sex with men

*We use the term "anal sex" to mean sex where one partner puts their penis into the other partner's anus, whether or not this ends in ejaculation.*



Brown JRG, *et al. Sex Transm Infect* 2023; 99:1–13. doi: 10.1136/sextrans-2022-055429

When did you **last** have anal sex with a man (either with or without a condom)?

- Between 23 March 2020 (first national lockdown) and December 2020
- Before 23 March 2020 (first national lockdown)
- Never

When did you **last** have condomless anal sex with a man?

- Between 23 March 2020 (first national lockdown) and December 2020
- Before 23 March 2020 (first national lockdown)
- Never

When did you **last** have condomless anal sex with a man?

- Before 23 March 2020 (first national lockdown)
- Never

Thinking now of the period **between 23 March 2020 (first national lockdown)** and **December 2020**, how many men did you have condomless anal sex with?

- 1
- 2
- 3
- 4
- 5
- 6
- 7
- 8
- 9
- 10
- 11-20
- 21-30
- 31-40
- 41-50

- More than 50

## About sex with women (including trans women)

*Please say when you last did something, even if this was not typical for you.*

Have you **ever** had sex of any kind with a woman [*please include any sexual contact, not just vaginal or anal intercourse*]?

- No
- Yes – virtual sex (e.g. phone sex, sexting)
- Yes – sex with physical contact (you could touch each other)
- Yes – virtual sex and sex with physical contact

## About sex with women (including trans women) <u>since December 2020</u>

Thinking of the time since December 2020, how many women have you had any kind of **physical sexual contact** with during this time [*please include any sexual contact, not just vaginal or anal intercourse*]?

- None
- 1
- 2
- 3
- 4
- 5
- 6
- 7
- 8
- 9
- 10
- 11-20
- 21-30
- 31-40



Brown JRG, *et al. Sex Transm Infect* 2023; 99:1–13. doi: 10.1136/sextrans-2022-055429

- 41-50
- More than 50

Was this woman a new sex partner (you had sex with her for the first time since December 2020)?

- No
- Yes

How many of these {number} women were new partners who you had sex with for the first time since December 2020?

- None
- 1
- 2
- 3
- 4
- 5
- 6
- 7
- 8
- 9
- 10
- 11-20
- 21-30
- 31-40
- 41-50
- More than 50

## About vaginal and anal sex with women (including trans women) <u>since December 2020</u>

*We use the term "vaginal sex" to mean sex where one partner puts their penis into the other partner's vagina and "anal sex" to mean sex where one partner puts their penis into the other partner's anus,*

*whether or not this ends in ejaculation.*

Thinking of the time since December 2020, have you had vaginal or anal sex with a woman?

- No
- Yes

Since December 2020, have you had condomless vaginal or anal sex with a woman?

- No
- Yes

Since December 2020, how many women have you had condomless vaginal or anal sex with?

- 1
- 2
- 3
- 4
- 5
- 6
- 7
- 8
- 9
- 10
- 11-20
- 21-30
- 31-40
- 41-50
- More than 50

Do you and this woman have the same HIV status?

- Yes, we have the same HIV status (both positive or both negative)
- No, one of us is positive and the other is negative
- I don’t know whether we have the same status or not

How many of these {number} women you had condomless vaginal or anal sex with did you know had the same HIV status as yourself (both positive or both negative)?

- I don’t know
- None
- 1
- 2
- 3
- 4
- 5
- 6
- 7
- 8
- 9
- 10
- 11-20
- 21-30
- 31-40
- 41-50
- More than 50

## About vaginal and anal sex with women (including trans women)

*We use the term "vaginal sex" to mean sex where one partner puts their penis into the other partner's vagina and "anal sex" to mean sex where one partner puts their penis into the other partner's anus, whether or not this ends in ejaculation.*

When did you **last** have vaginal or anal sex with a woman?

- Between 23 March 2020 (first national lockdown) and December 2020
- Before 23 March 2020 (first national lockdown)
- Never

When did you **last** have condomless vaginal or anal sex with a woman?

- Between 23 March 2020 (first national lockdown) and December 2020
- Before 23 March 2020 (first national lockdown)
- Never

When did you **last** have condomless vaginal or anal sex with a woman?

- Before 23 March 2020 (first national lockdown)
- Never

Thinking of the period between **23 March 2020 (first national lockdown)** and **December 2020**, how many women did you have condomless vaginal or anal sex with?

- None
- 1
- 2
- 3
- 4
- 5
- 6
- 7
- 8
- 9
- 10
- 11-20
- 21-30

- 31-40
- 41-50
- More than 50

## About drugs and sex

Have you **ever** taken crystal meth, mephedrone or GHB/GBL?

- No
- Yes

When did you **last** take crystal meth, mephedrone or GHB/GBL?

- Since December 2020
- Between 23 March 2020 (first national lockdown) and December 2020
- Before 23 March 2020 (first national lockdown)

How much of the sex you have had was after taking crystal meth and/or mephedrone and/or GHB/GBL?

| | None of it | A little | Less than half | About half | More than half | Almost all | All of it | I don’t know |
|---|---|---|---|---|---|---|---|---|
| Since December 2020 | | | | | | | | |
| Between 23 March 2020 (first national lockdown) and December 2020 | | | | | | | | |

## About injecting drugs

Have you **ever** injected any drug other than medicines or anabolic steroids, or had someone inject these for you?

- No
- Yes

When did you **last** inject any drug other than anabolic steroids or medicines, or had someone inject these for you?

- Since December 2020
- Between 23 March 2020 (first national lockdown) and December 2020
- Before 23 March 2020 (first national lockdown)

## About your well-being

Overall, how satisfied are you with your life nowadays?

- Not at all – 0
- 1
- 2
- 3
- 4
- 5
- 6
- 7
- 8
- 9
- Completely - 10

Overall, to what extent do you feel the things you do in your life are worthwhile?

- Not at all – 0
- 1
- 2
- 3
- 4
- 5
- 6
- 7
- 8
- 9
- Completely - 10

Overall, how happy did you feel yesterday?

- Not at all – 0
- 1
- 2
- 3

- 4
- 5
- 6
- 7
- 8
- 9
- Completely – 10

Overall, how anxious did you feel yesterday?

- Not at all – 0
- 1
- 2
- 3
- 4
- 5
- 6
- 7
- 8
- 9
- Completely - 10

## About your experience of COVID-19

Have you experienced any of these symptoms since **December 2020**? [*tick all that apply*]

- High temperature
- A new continuous cough
- Loss or changed sense of normal smell or taste
- Shortness of breath or trouble breathing
- Runny or stuffy nose
- Muscle or body aches
- Headaches
- Sore throat
- Fatigue

- Diarrhoea/Digestive issues/Upset stomach
- None of these

Are you **currently** experiencing any of these symptoms? [*tick all that apply*]

- High temperature
- A new continuous cough
- Loss or changed sense of normal smell or taste
- Shortness of breath or trouble breathing
- Runny or stuffy nose Muscle or body aches
- Headaches
- Sore throat
- Fatigue
- Diarrhoea/Digestive issues/Upset stomach
- None of these

Have you had a throat and nose swab to see if you had coronavirus? [*an antigen test to see if you had coronavirus at the time of the test*]

- No
- Yes

{if yes} How many coronavirus swab tests have you had?

- 1
- 2
- 3
- 4
- 5 or more

{if 1} Was the result of your coronavirus swab test positive (showing you had coronavirus at the time of the test)?

- No
- Yes
- Still waiting for result

{if yes} When did you have a positive result from a coronavirus swab test?

- December 2019
- January 2020
- February 2020
- March 2020
- April 2020
- May 2020
- June 2020
- July 2020
- August 2020
- September 2020
- October 2020
- November 2020
- December 2020
- January 2021
- February 2021
- March 2021
- April 2021

{if 2 or more} Have you had a positive result from any of your coronavirus swab tests (showing you had coronavirus at the time of the test)?

- No
- Yes
- Still waiting for results

{if yes} When did you have a positive result from a coronavirus swab test? [*tick all that apply*]

- December 2019
- January 2020
- February 2020
- March 2020
- April 2020
- May 2020
- June 2020

- July 2020
- August 2020
- September 2020
- October 2020
- November 2020
- December 2020
- January 2021
- February 2021
- March 2021
- April 2021

Have you had a blood test to see if you previously had coronavirus? [*an antibody test*]

- No
- Yes

{if yes} How many coronavirus antibody blood tests have you had?

- 1
- 2
- 3
- 4
- 5 or more

{if 1} Was the result of your coronavirus antibody test positive (showing you had coronavirus previously)?

- No
- Yes
- Still waiting for result

{if yes} When did you have a positive result from a coronavirus antibody test?

- December 2019
- January 2020
- February 2020

- March 2020
- April 2020
- May 2020
- June 2020
- July 2020
- August 2020
- September 2020
- October 2020
- November 2020
- December 2020
- January 2021
- February 2021
- March 2021
- April 2021

{if 2 or more} Have you had a positive result from any of your coronavirus antibody tests (showing you had coronavirus previously)?

- No
- Yes
- Still waiting for results

{if yes} When did you have a positive result from a coronavirus antibody test? [*tick all that apply*]

- December 2019
- January 2020
- February 2020
- March 2020
- April 2020
- May 2020
- June 2020
- July 2020
- August 2020

- September 2020
- October 2020
- November 2020
- December 2020
- January 2021
- February 2021
- March 2021
- April 2021

Have you received a vaccine for the coronavirus (COVID-19)?

- Yes, I have received my first vaccine dose
- Yes, I have received both vaccine doses
- No
- Don't know
- Prefer not to say.

Have you been offered the vaccine for the coronavirus (COVID-19)?

- Yes, I am waiting to be vaccinated
- Yes, I have been offered the vaccine and I have decided not to be vaccinated
- No
- Don't know
- Prefer not to say.

Have you been admitted to hospital because of coronavirus symptoms?

- No
- Yes

How many nights did you stay in hospital because of coronavirus symptoms?

- I am still in hospital



Brown JRG, *et al*. *Sex Transm Infect* 2023; 99:1–13. doi: 10.1136/sextrans-2022-055429

- I did not stay overnight

Number of nights:

Were you taken to intensive care because of coronavirus symptoms?

- No
- Yes

Do you have any of the medical conditions identified as placing someone at greater risk of severe illness from COVID-19 [*click here for information about these conditions*]?

- No
- Yes

## Just before you submit

**Your privacy is important.** This survey is completely anonymous and confidential. We do not collect any information about you that would allow anybody to identify you.

You may have completed our first survey in summer 2020 and/or our second survey in November/December 2020. To help us link your anonymous responses to past surveys, please consider providing the following information which is personal to you but not easily guessed:

A memorable female name (for example, your mother's or a celebrity name):

The name of a street where you lived as a child:

**Now please submit.** You will then see some links to information on sexual health and support services. The information you have given will help plan better sexual health services.



Brown JRG, *et al. Sex Transm Infect* 2023; 99:1–13. doi: 10.1136/sextrans-2022-055429
